# Enhancement of Leaf Gas Exchange and Primary Metabolites under Carbon Dioxide Enrichment Up-Regulates the Production of Secondary Metabolites in *Labisia pumila* Seedlings 

**DOI:** 10.3390/molecules16053761

**Published:** 2011-05-04

**Authors:** Mohd Hafiz Ibrahim, Hawa Z.E. Jaafar

**Affiliations:** Department of Crop Science, Faculty of Agriculture, University Putra Malaysia, 43400 Serdang, Selangor, Malaysia; Email: mhafizphd@gmail.com

**Keywords:** elevated CO_2_, photosynthesis, maximum quantum efficiency of photosystem II (f_v_/f_m_), medicinal herb Kacip Fatimah, total non structural carbohydrates

## Abstract

A split plot 3 by 3 experiment was designed to investigate and distinguish the relationships among production of primary metabolites (soluble sugar and starch), secondary metabolites (total phenolics, TP; total flavonoids, TF) and leaf gas exchange of three varieties of the Malaysian medicinal herb *Labisia pumila* Blume, namely the varieties *alata*, *pumila* and *lanceolata*, under three levels of CO_2_ enrichment (400, 800 and 1,200 µmol mol^−1^) for 15 weeks. The treatment effects were solely contributed by CO_2_ enrichment levels; no varietal differences were observed. As CO_2_ levels increased from 400 to 1,200 µmol mol^−1^, the production of carbohydrates also increased steadily, especially for starch more than soluble sugar (sucrose). TF and TP content, simultaneously, reached their peaks under 1,200 µmol exposure, followed by 800 and 400 µmol mol^−1^. Net photosynthesis (A) and quantum efficiency of photosystem II (f_v_/f_m_) were also enhanced as CO_2_ increased from 400 to 1,200 µmol mol^−1^. Leaf gas exchange characteristics displayed a significant positive relationship with the production of secondary metabolites and carbohydrate contents. The increase in production of TP and TFs were manifested by high C/N ratio and low protein content in *L. pumila* seedlings, and accompanied by reduction in cholorophyll content that exhibited very significant negative relationships with total soluble sugar, starch and total non structural carbohydrate.

## 1. Introduction

The steady increase in atmospheric CO_2_ concentration due to climate change and/or agricultural practices is likely to affect biota by producing changes, not only in plant growth and allocation, but also in plant tissue chemical composition [1]. Among such composition changes, most source-sink hypotheses (carbon nutrient balance hypothesis [2] and growth-differentiation balance hypothesis [3]) assume that elevated CO_2_ concentration promotes a relative increase in carbon availability that is accumulated in total non structural carbohydrate (TNC) and carbon based secondary metabolites (CBSM) when the provided carbon amounts exceed growth requirements [4]. The allocation of carbon to growth for differentiation should, by competition for an internal resource of limited availability, diminish the allocation of carbon to secondary metabolism. The differentiation process is, however, a prequisite for the full development of secondary metabolism because differentiation produces the tissue and cells in which secondary products and the corresponding biosynthetic enzymes are formed [5]. 

Secondary metabolism is linked to primary metabolism by the rates at which substrates are diverted from primary pathways and funneled into the secondary biosynthetic routes. Due to these, several environmental factors affecting growth, photosynthesis and other parts of primary metabolism will also affect secondary metabolism [2]. These hypotheses predict a larger accumulation of carbon based secondary metabolites such as phenolics, terpenes or structural carbohydrates at elevated CO_2_. When protein synthesis is restricted under high carbon-nitrogen ratio availability, the consequent lower demand of amino acids could determine the stimulation of phenolics compounds synthesis [6]. The changes in CO_2_ concentrations could have a significant consequence for ecosystem functioning and plant litter decomposition [7]. The increase in CBSM can occur in natural plant ecosystem, but may be created deliberately by CO_2_ enrichment techniques in controlled environment system to increase the production of some plants secondary metabolites compounds [8,9]. Under high CO_2_, the levels of non nitrogenous metabolites derived from the shikimic acid pathway such as phenolics acid, lignin, hydrolysable tannins and proanthocyanidins, usually increase [10]. The increase in CBSM frequently occurs when environmental conditions promote the accumulation of TNC in plants. The increase in atmospheric CO_2_ concentration often increases total non structural carbohydrate (TNC) concentrations in plants that possibly stimulate the production of secondary metabolites [11].

Flavonoids and phenolics are the most important groups of secondary metabolites and bioactive compounds in plants [12]. High content of natural flavonoids and phenolics acids are found in green tea, fruits, and vegetable, while some amounts of phenolics also exist in red wine and coffee [13,14]. They are also a kind of natural products and antioxidant substances capable of scavenging free superoxide radicals, anti-aging effects and reducing the risk of cancer. Phenolic and flavonoid compounds are also well known as one of the major contributors to the antioxidant activity of herbs and medicinal plants [15,16]. It was discovered that these secondary metabolites function to reduce blood lipid glucose and enhance human immunity [17]. Their function in human health is supported by the ability to induce human protective enzyme systems, and by number of epidemiological studies suggesting protective effects against cardiovascular disease, cancers and other related diseases [18]. 

The enrichment of high CO_2_ can enhance the medicinal properties of some medicinal plants such as *Labisia pumila* Blume, locally known in Malaysia as Kacip Fatimah. It is a sub-herbaceous plant with creeping stems from the family Myrsinaceae. It is found widespread in Indochina and throughout the Malaysian forest [19]. Traditionally *L. pumila* has been used by Malay women to induce and facilitate childbirth, as well as a post-partum medicine [20]. The other uses of this herb are as treatments for dysentery, dysmenorrhea, flatulence and gonorrhea [21]. Recently, it was found that the bioactive compounds of *L. pumila* consisted of resorcinols, flavonoids and phenolic acids [19,22], which have been shown to have high antioxidant properties [23]. Recently, the enrichment of *L*. *pumila* with high levels of CO_2_ was found to increase the secondary metabolite production (phenolics and flavonoids) of this plant [24]. A similar result was also observed in ginger (*Zingiber officianale*) [25].

Although CBSM constitutes a significant sink for assimilated carbon, to date, there is no clear picture on how these compounds respond to different levels of elevated CO_2_ especially for herbal plants like *L. pumila*. Many studies have investigated the effects of elevated CO_2_ on plant primary metabolism, but relatively few studies have investigated the response of plant CBSM to increasing CO_2_. The main purpose of this study was to investigate the effects of three levels of carbon dioxide enrichment (400, 800 and 1,200 µmol mol^−1^ CO_2_) on three varieties of *L. pumila* (var. *alata,* var. *pumila* and var. *lanceolata*) on alterations of secondary metabolites (total flavonoid and phenolics), chlorophyll content, TNC (starch plus sugars), net photosynthesis, quantum efficiency, C/N ratio and proteins content. The relationships among these parameters were also determined.

## 2. Results and Discussion

### 2.1. Total Flavonoids and Phenolics Contents, and Their Profiling

Carbon dioxide levels had a significant (*P* ≤ 0.05) impact on the production of total phenolics and flavonoid production (Table 1). As CO_2_ levels increased from 400 to 1,200 µmol mol^−1^ CO_2_ more total phenolics and flavonoids were produced. *L. pumila* Blume partitioned more of the secondary metabolites to the leaves, followed by the roots and then stems. In leaf, total flavonoids was enhanced by 80% and 95%, respectively, in 800 and 1,200 µmol mol^−1^ compared to 400 µmol mol^−1^ CO_2_. Total phenolics, on the other hand, increased by 30%-58% under elevated CO_2_ compared to the ambient CO_2_. Increases in rutin and gallic acid in *L. pumila* seedlings enriched with high CO_2_ might increase the medicinal properties of this plant. Previous studies have shown that rutin and gallic acid act as free radical scavengers and as inducers of differentiation and apoptosis in leukemia, lung cancer, and colon adenocarcinoma cell lines, as well as in normal lympochyte cells [26,27]. The enhancement of total plant flavonoids and phenolics usually occurred when plants are deficient in nitrogen [28,29]. This improvement in plant secondary metabolites might be due to increased total non structural carbohydrates (TNC) as exhibited by the correlation coefficient (r^2^ = 0.77; Table 2), although a higher correlation coefficient (r^2^ = 0.98) was displayed by total soluble sugar implying that the accumulation of soluble sugar could be more responsible for the up regulation of plant secondary metabolite production.

The current result was in agreement with Amin *et al.* [30] who proposed that the increase in secondary metabolites (flavonoids) content was due to an increase in total soluble sugar as observed in onion with increases of the former by 7% as a result of the latter’s enhancement by 21%. Increases in total flavonoids and phenolics compounds under elevated CO_2_ were also reported by Wang *et al.* [31] and Sttute *et al.* [32].

**Table 1:** Accumulation and partitioning of total flavonoids (TF) and total phenolics (TP) in different plant parts of *Labisia pumila* Blume under different CO_2_ levels.

CO_2_ levels	Plant parts	Total flavonoid	Total phenolics
(µmol mol^−1^)		(mg g^−1^ rutin dry weight)	(mg g^-1^ Gallic acid dry weight)
400	Leaf	0.126 ± 0.018a	0.815 ± 0.017a
	Stem	0.081 ± 0.022b	0.511 ± 0.022b
	Root	0.062 ± 0.032c	0.321 ± 0.018c
800	Leaf	0.227 ± 0.017a	1.067 ± 0.023a
	Stem	0.133 ± 0.023b	0.778 ± 0.021b
	Root	0.087 ± 0.024c	0.443 ± 0.011c
1,200	Leaf	0.246 ± 0.021a	1.289 ± 0.032a
	Stem	0.145 ± 0.032b	0.872 ± 0.027b
	Root	0.095 ± 0.031c	0.554 ± 0.041c

All analyses are mean ± standard error of mean (SEM). N = 15. Means not sharing a common letter were significantly different at P ≤ 0.05.

**Table 2:** Correlations among the measured parameters in the experiments.

Parameters	1	2	3	4	5	6	7	8	9	10	11	12	13	14	15	16
**1. Flavonoid**	1.00															
**2. Phenolics**	0.97*	1.00														
**3. TSS**	0.98*	0.98*	1.00													
**4. Starch**	0.87*	0.82*	0.80*	1.00												
**5. TNC**	0.77*	0.76*	0.76*	0.77*	1.00											
**6. Photosynthesis**	0.87*	0.87*	0.86*	0.78*	0.77*	1.00										
**7. fv/fm**	0.77*	0.67	0.04	0.56	0.08	0.77*	1.00									
**8. Nitrogen**	−0.87*	−0.92*	0.43	0.06	0.05	0.87*	0.13	1.00								
**9. Carbon**	0.56	0.46	0.54	0.45	0.56	0.77*	0.01	−0.12	1.00							
**10. C/N**	0.78*	0.77*	0.53	0.57	0.44	0.67	0.21	0.21	0.76*	1.00						
**11. Chlorophyll a**	−0.76*	−0.77*	0.44	0.33	0.34	−0.33	0.03	0.01	0.32	0.07	1.00					
**12. Chlorophyll b**	−0.75*	−0.88*	0.43	0.23	0.32	−0.32	0.09	0.07	0.21	0.08	0.88*	1.00				
**13.T. Chlorophyll**	−0.78*	−0.76*	0.54	0.21	0.36	−0.21	0.12	0.12	0.18	0.16	0.89*	0.88*	1.00			
**14. T. biomass**	0.88*	0.76*	0.65	0.57*	0.67*	0.87*	0.32	0.78	0.75*	0.23	−0.78*	−0.66*	−0.89*	1.00		
**15. SLA**	0.87*	0.78*	0.05	0.41	0.12	0.86*	0.07	0.65	0.55	0.44	0.33	0.32	0.91*	0.87*	1.00	
**16. Protein**	−0.87*	−0.76*	0.07	0.09	0.03	−0.76*	0.21	−0.65*	−0.76*	−0.78*	0.78*	0.56*	−0.87*	−0.89*	−0.54	1.00

* and ** respectively significant at *P* ≤ 0.05 or *P* ≤ 0.01.

### 2.2. Total Soluble Sugar, Starch and Total Non Structurable Carbohydrate (TNC) and Their Profiling

The accumulation and partitioning of carbohydrates were influenced by carbon dioxide enrichment of *L. pumila* (*P* ≤ 0.05). The accumulation of carbohydrates in different parts of the plant followed a descending order of leaf > root > stem. As CO_2_ enrichment levels increased, the concentration of total soluble sugar, starch and TNC also increased (Table 3). The concentration of sucrose and starch registered the lowest values under 400 µmol mol^−1^ CO_2_ ennrichment, compared to the plants under higher CO_2_ exposure. Under ambient conditions lower sucrose and starch were produced in the leaf, stem and root compared to those plants exposed to high CO_2_ concentration. In all plant parts of *L. pumila*, the increase in starch content was larger than the increase in sugar concentration [33]. Results thus suggested that CO_2_ enrichment of *L. pumila* under high CO_2_ was able to enhance the soluble sugar and starch contents, which had simultaneously enhanced the TNC. Similar observation was found by other researchers [34,35,36,37]. 

The accumulation of carbohydrate in CO_2_-enriched plant might be attributed to dilution of plant tissue nitrogen in enhanced plant growth under elevated CO_2_, especially, when nitrogen is limited; this could reduce sink size of the plant, hence, reducing the translocation of carbohydrates to other plant parts [38]. The extra carbohydrates accumulated in *L. pumila* plants might be channeled to the production of secondary metabolites (total phenols and flavonoids), thus explaining why the production of secondary metabolites was up-regulated in enriched plants. Carbohydrates are basic compounds required to produce phenolic compounds through the shikimic acid pathway where extra carbohydrates derived from glycolisis and the pentose phosphate pathway are converted into aromatic amino acids [2]. Previous studies by Shui *et al.* [39] showed that an increase in secondary metabolites was related to the balance between carbohydrate source and sink; the greater the source-sink ratio, the greater the production of secondary metabolites that might occur.

**Table 3:** Accumulation and partitioning of total soluble sugar (TSS), starch and total non structurable carbohydrate (TNC) in different plant parts of *Labisia pumila* Blume. under different CO_2_ levels.

CO_2_ levels	Plant parts	TSS (mg g^−1^ sucrose dry weight)	Starch (mg g^−1^ glucose dry weight)	TNC (mg g^−1^ dry weight)
(µmol mol^−1^)				
400	Leaf	15.10 ± 0.65a	40.34 ± 0.98a	56.14 ± 2.31a
	Stem	12.32 ± 0.77b	30.24 ± 0.87b	42.57 ± 1.16b
	Root	10.34 ± 0.87c	24.34 ± 0.66c	34.68 ± 1.09c
800	Leaf	25.10 ± 0.56a	56.67 ± 0.67a	81.65 ± 0.99a
	Stem	18.76 ± 0.44b	45.67 ± 0.44b	65.78 ± 0.78b
	Root	15.32 ± 0.76c	32.16 ± 0.57c	47.67 ± 1.45c
1,200	Leaf	26.97 ± 0.76a	70.24 ± 0.55a	97.21 ± 3.21a
	Stem	19.45 ± 0.56b	60.54 ± 0.45b	79.89 ± 4.32b
	Root	15.43 ± 0.43c	45.67 ± 0.32c	62.10 ± 4.12c

All analyses are mean ± standard error of mean (SEM), N = 15. Means not sharing a common single letter were significantly different at P ≤ 0.05.

### 2.3. Photosynthesis and Maximum Quantum Efficiency of Photosystem II (Fv/Fm)

The photosynthesis rate and f_v_/f_m_ ratio was influenced by CO_2_ levels (*P* ≤ 0.05; Table 4); however, no varietal differences were observed. Leaf net photosynthesis and f_v_/f_m_ rate increased with increasing CO_2_ fertilization in an ascending order 400 > 800 > 1,200 µmol mol^−1^ CO_2_. 

**Table 4:** Effects of different nitrogen levels on some physiological parameters in *L. pumila* Blume under CO_2_ enrichment.

Parameters	400 µmol mol^−1^	800 µmol mol^−1^	1,200 µmol mol^−1^
Net photosynthesis	5.73 ± 0.56c	11.10 ± 0.44b	13.01 ± 0.67a
f_v_/f_m_	0.76 ± 0.02c	0.89 ± 0.12b	0.93 ± 0.07a
Leaf nitrogen	2.96 ± 0.17a	1.63 ± 0.06b	0.97 ± 0.01c
Leaf carbon	27.64 ± 1.23c	32.14 ± 1.09b	34.12 ± 1.78a
C/N	9.33 ± 0.77c	19.07 ± 0.98b	34.55 ± 2.34a
Chlorophyll a	6.16 ± 0.76a	3.12 ± 0.11b	2.97 ± 0.56c
Chlorophyll b	17.36 ± 0.12a	12.76 ± 0.32b	11.76 ± 0.21c
Chlorophyll a+b	24.22 ± 1.23a	16.13 ± 0.97b	12.89 ± 0.88c
Total biomass	10.78 ± 1.23c	16.04 ± 0.76b	17.48 ± 0.22a
Specific leaf area	48.90 ± 2.23a	42.32 ± 1.23b	39.87 ± 1.23c
Protein content	7.17 ± 1.23a	6.06 ± 0.98b	4.17 ± 2.31c

All analyses are mean ± standard error of mean (SEM), N = 15. Means not sharing a common single letter were significantly different at P ≤ 0.05.

The highest net photosynthesis was obtained in *L. pumila* exposed to CO_2_ 1,200 µmol mol^−1^ (13.01 µmol m^−2^ s^−1^), followed with 800 µmol mol^−1^ (11.10 µmol m^−2^ s^−1^) and 400 µmol mol^−1^ CO_2_ (5.73 µmol m^−2^ s^−1^). The f_v_/f_m_ ratio was 17% and 22% higher in 800 and 1,200 µmol mol^−1^ CO_2_, respectively, compared to the ambient CO_2_ level. The findings exhibited the importance of CO_2_ in further enhancing properties of leaf gas exchange and photosystem II efficiency of *L. pumila* plants exposed to CO_2_ enrichment. 

The increase in photosynthesis and photosystem II efficiency in the present work could have stimulated the production of plant secondary metabolites, as shown by the positive correlation coefficients in Table 2 between photosynthesis and secondary metabolites (r^2^ = 0.87*) of total phenolics and flavonoids, and photosystem II efficiency and total flavonoids (r^2^ = 0.77*). A possible explanation for this might be that the increase in photosynthetic rate could have increased the shikimic acid pathway that enhanced the production of plant secondary metabolites, and this is due in turn to an increase in the concentration of soluble sugar [40,41]. Some studies reported that when production of secondary metabolites increased, the photosynthesis would decrease due to feedback control of the secondary metabolites production [42], however, such an effect was not observed in the present study.

### 2.4. Leaf Nitrogen and Carbon to Nitrogen Ratio (C:N)

The increase in CO_2_ levels significantly reduced the leaf nitrogen content (*P* ≤ 0.05; Table 4). As the CO_2_ enrichment levels increased from 400 to 1,200 µmol mol^−1^ CO_2_ leaf tissue nitrogen also decreased considerably. Leaf nitrogen content in 400 µmol mol^−1^ CO_2_ was 44% and 67% higher than those in 800 and 1,200 µmol mol^−1^ CO_2_, respectively. The decrease in leaf tissue nitrogen might result from diminished of nitrate content in the leaf that signified the enhanced nitrate assimilation of plant under elevated CO_2_ [43]. Concurrently, the increase in CO_2_ levels lead to an increase in plant C/N ratio under high CO_2_ fertilization. The C/N ratio of 400 µmol mol^−1^ CO_2_ was 9.33, compared to those of 800 and 1,200 µmol mol^−1^ CO_2_ that registered increasing values at 19.07 and 34.55, respectively. A similar increase in C/N ratio of plants enriched with high CO_2_ was also observed by Fonseca *et al.* in *Plantago major* [37]. High C:N ratio had a significant positive relationship (*P* ≤ 0.01) with total flavonoids and phenolics compounds (r^2^ = 0.78; Table 2) signifying a good direct association between the C:N ratio and plant secondary metabolites. Correspondingly, the C/N ratio displayed a significant positive relationship with photosynthesis (r^2^ = 0.67), implying that increase in C/N ratio would increase the photosynthetic capacity of *L. pumila*. In the present study, the increase in C/N ratio had also enhanced the photosynthetic capacity of *L. pumila* seedlings, and this suggested an enhanced synthesis of plant secondary metabolites, especially the flavonoids and phenolics [44].

### 2.5. Chlorophyll Content

Chlorophyll content was influenced by the application of CO_2_ levels to the seedlings (*P* ≤ 0.01; Table 4). As the levels of CO_2_ increased from 400 to 1,200 µmol mol^−1^ CO_2_, chlorophyll a, b and total chlorophyll a+b were reduced. The decrease in chlorophyll content with increasing CO_2_ levels has been reported by Porteus *et al.* [45]. It was found from the correlation (Table 2) that chlorophyll a, b and total were significantly (*P* ≤ 0.01) and negatively related with secondary metabolites. Competition between secondary metabolites and chlorophyll content fits well with the prediction of the protein competition model (PCM) that the secondary metabolites content is controlled by the competition between protein and secondary metabolites biosynthesis pathway and its metabolites regulation. The negative relationship between the secondary metabolites and chlorophyll is a sign of gradual switch of investment from protein to polyphenols production [46]. The same discovery was also obtained by Michel *et al.* [47] for flavonoid and chlorophyll content in *Arabidopsis*, which suggested that the production of secondary metabolites was competing with light harvesting protein when nitrogen content in leaf was low.

### 2.6. Plant Biomass and Specific Leaf Area

Different CO_2_ levels significantly (*P* ≤ 0.01) affected plant biomass and specific leaf area (SLA) of *L. pumila* seedlings (Table 4). With increasing CO_2_ concentration, plant biomass and specific leaf area increased significantly. A high content of plant biomass (17.48 g) was observed in 1,200 µmol mol^−^^1^ CO_2_ followed by 800 µmol mol^−^^1^ CO_2_ (16.04 g) and 400 µmol mol^−^^1^ CO_2_ (10.78 g). It was also observed that the leaf thickness was enhanced under elevated CO_2_. Plant enriched with high CO_2_ have 13.5 to 18.5% more thicker leaf than the plant exposed to ambient CO_2_ concentration. The enhanced total plant biomass might due to increase in photosynthetic rate [48] as supported by the positive correlation (r^2^ = 0.87) between plant biomass and photosynthetic rate was. The increase in secondary metabolites under high CO_2_ might also be related to increased thickness of the leaf that contained thick mesophyll cell layer [49]. As in improved total biomass and SLA, Nagasubramaniam *et al.* [50] and Jeyakumar *et al.* [40] also reported of the enhanced plant height, leaf area, plant biomass and growth rate as a result of exposure to high CO_2_, which could be directly involved with salicylic acid content (phenolics compound).

### 2.7. Protein Content

Soluble protein of *L. pumila* was influenced by the CO_2_ levels (*P* ≤ 0.01; Table 4). As the levels of CO_2_ increased from 400 to 1,200 µmol mol^−^^1^ the soluble protein decreased. The highest protein content was obtained in 400 µmol mol^−1^ CO_2_ (7.17 mg g^−1^ dry weight) and the lowest was obtained in 1,200 µmol mol^−1^ CO_2_ (4.17 mg g^−1^ dry weight). Similar result as in the present study was also observed by Gleadow *et al.* [51] in cassava where the highest protein accumulation was found under ambient CO_2_. Protein content was also observed to have a negative relationship with total phenols and flavonoid (r^2^ = −0.87; R^2^ = −0.76) indicating that the up-regulation of plant secondary metabolites might occur when protein content was reduced [52]. Decrease in protein production under high CO_2_ and low nitrogen levels, as exhibited by the present work, might decrease the usage of PAL in protein synthesis, hence channeling it for the biosynthesis of plant secondary metabolites [53]. This explains why increase in secondary metabolites might be up-regulated under the condition of high CO_2_ and low nitrogen. 

## 3. Experimental Section

### 3.1. Experimental Location, Plant Materials and Treatments

The experiment was carried out under a growth house at Field 2, Faculty of Agriculture Glasshouse Complex, Universiti Putra Malaysia (longitude 101°44’N and latitude 2°58’S, 68 m above sea level) with a mean atmospheric pressure of 1.013 kPa. Three-month old *L. pumila* seedlings of var *alata,* var *pumila* and var *lanceolata* were left for a month in a nursery to acclimatize until they were ready for the treatments. Carbon dioxide enrichment treatment started when the seedlings reached 4 months of age where plants were exposed to 400, 800 and 1,200 µmol^−1^ mol^−1^ CO_2_. This 2-factorial experiment was arranged in a split plot using a randomized complete block design with CO_2_ levels being the main plot, and varieties as the sub-plot replicated three times. Each treatment consisted of ten seedlings. 

### 3.2. Growth House Microclimate and CO_2_ Enrichment Treatment

The seedlings were raised in specially constructed growth houses receiving 12-h photoperiod and average photosynthetic photon flux density of 300 µmol m^−2^ s^−1^. Day and night temperatures were recorded at 30 ± 1.0 °C and 20 ± 1.5 °C, respectively, and relative humidity at about 70% to 80%. Vapor pressure deficit ranged from 1.01 to 2.52 kPa. Carbon dioxide at 99.8% purity was supplied from a high–pressure CO_2_ cylinder and injected through a pressure regulator into fully sealed 2 m × 3 m growth houses at 2-h daily and applied continuous from 08:00 to 10:00 a.m. [54]. The CO_2_ concentration at different treatments was measured using Air Sense ™ CO_2_ sensors designated to each chamber during CO_2_ exposition period. Plants were watered three to four times a day at 5 min per session to ensure normal growth of plant using drip irrigation with emitter capacity of 2 L h^−1^. The experiment lasted for 15 weeks from the onset of treatment.

### 3.3. Total Phenolics and Total Flavonoids Quantification

The method of extraction and quantification for total phenolics and flavonoids contents followed after Jaafar *et al.* [24]. An amount of ground tissue sample (0.1 g) was extracted with 80% ethanol (10 mL) on an orbital shaker for 120 minutes at 50 °C. The mixture was subsequently filtered (Whatman™ No.1), and the filtrate was used for the quantification of total phenolics and total flavonoids. Folin–Ciocalteu reagent (diluted 10-fold) was used to determine the total phenolics content of the leaf samples. Two hundred µL of the sample extract was mixed with Follin–Ciocalteau reagent (1.5 mL) and allowed to stand at 22 °C for 5 minutes before adding NaNO_3_ solution (1.5 mL, 60 g L^−1^). After two hours at 22 °C, absorbance was measured at 725 nm. The results were expressed as mg g^−1^ gallic acid equivalent (mg GAE/g dry sample).For total flavonoids determination, sample (1 mL) was mixed with NaNO_3_ (0.3 mL) in a test tube covered with aluminium foil, and left for 5 minutes. Then 10% AlCl_3_ (0.3 mL) was added followed by addition of 1 M NaOH (2 mL) and the absorbance was measured at 510 nm using rutin as a standard (mg rutin g^−1^ dry sample). 

### 3.4. Soluble Carbohydrates

Soluble carbohydrates were measured spectrophotometrically using the method described by Edward [55]. Samples (0.5 g) were placed in 15 mL conical tubes. Then distilled water (10 mL) was added and the mixture was then vortexed and incubated for 10 minutes. Anthrone reagent was prepared using anthrone (0.1 g) that was dissolved in 95% sulphuric acid (50 mL). Sucrose was used as a standard stock solution to prepare a standard curve for the quantification of sucrose in the sample. The mixed sample of ground dry sample and distilled water was centrifuged at a speed of 3,400 rpm for 10 minutes and then filtered to get the supernatant. To an aliquot (4 mL) of the sample was added anthrone reagent (8 mL) and the mixture was placed in a waterbath set at 100 °C for 5 minutes before the sample was measured at absorbance 620 nm using UV160U spectrophotometer (Shimadzu, Japan). The soluble sugar in the sample was expresses as mg sucrose g^−^^1^ dry sample.

### 3.5. Starch Determination

Starch content was determined spectrophometrically using method by Thayumanavam and Sadasivam [56]. In this method, dry sample (about 0.5 g) was homogenized in hot 80% ethanol (10 mL) to remove the sugar. The sample was then centrifuged at 5,000 rpm for 5 minutes and then the residue was retained. After that, distilled water (5.0 mL) and 52% perchloric acid (6.5 mL) were added to the residue, then the solution was centrifuged and the supernatant separated and then filtered through no. 5 filter paper (Whatman). The processes were repeated until the supernatant was made up to 100 mL. An aliquot of the supernatant (100 µL) was added to distilled water until the volume became 1 mL. After that, anthrone reagent (Sigma, USA; 4 mL, prepared with 95% sulphuric acid by adding 2 g of anthrone to 100 mL 95% sulphuric acid) was added to a tube. The mixed solution was placed in the water bath at 100 °C for eight minutes and then cooled to the temperature room, and then the sample was read at absorbance of 630 nm to determine the sample starch content. Glucose was used as a standard and starch content was expressed as mg glucose equivalent g^−^^1^ dry sample. 

### 3.6. Total Non Structural Carbohydrate (TNC)

The total non structural carbohydrate was calculated as the sum of total soluble sugar and starch content [57].

### 3.7. Photosynthesis Rate

The measurement was obtained from a closed infra-red gas analyzer LICOR 6400 Portable Photosynthesis System (IRGA, Licor Inc. NE, USA). Prior to use, the instrument was warmed for 30 minutes and calibrated with the ZERO IRGA mode. Two steps are required in the calibration process: first, the initial zeroing process for the built-in flow meter; and second, zeroing process for the infra-red gas analyzer. The measurements used optimal conditions set by Jaafar *et al.* [58] of 400 µmol mol^−1^ CO_2_ 30 °C cuvette temperature, 60% relative humidity with air flow rate set at 500 cm^3^ min^−1^, and modified cuvette condition of 800 µmol m^−2^ s^−1^ photosynthetically photon flux density (PPFD). The measurements of gas exchange were carried out between 09:00 to 11:00 a.m. using fully expanded young leaves numbered three and four from plant apex to record net photosynthesis rate (A). The operation was automatic and the data were stored in the LI-6400 console and analyzed by “*Photosyn Assistant*” software (Version 3, Lincoln Inc, USA). Several precautions were taken to avoid errors during measurements. Leaf surfaces were cleaned and dried using tissue paper before enclosed in the leaf cuvette.

### 3.8. Maximum Quantum Efficiency of Photosystem II (fv/fm)

Measurements of chlorophyll fluorescence were taken from fully expanded leaf of the second leaves. Leaves were darkened for 15 minutes by attaching light-exclusion clips to the central region of the leaf surface. Chlorophyll fluorescence was measured using a portable chlorophyll fluorescence meter (Handy PEA, Hansatech Instruments Ltd, Kings Lynn, UK). Measurements were recorded up for 5 seconds [59]. The fluorescence responses were induced by emitting diodes. Measurement of f_o_ (initial fluorescence), f_M_ (maximum fluorescence) and f_V_ (variable fluorescence) were obtained from this procedure. f_V_ is derived as the differences between f_M_ and f_o_. The mean value of three representative plants was used to represent each sub-plot.

### 3.9. Total Carbon, Nitrogen and C:N Ratio

Total carbon and C:N ratio were measured by using a CNS 2000 analyzer (Model A Analyst 300, LECO Inc, USA). This was performed by placing ground leaf sample (0.05 g) into the combustion boat. Successively, the combustion boat was transferred to the loader before the sample was burned at 1,350 °C to obtain the reading of total carbon and nitrogen content of the samples. 

### 3.10. Chlorophyll Content

Total chlorophyll content was measured by method from Idso *et al.* [60] using fresh weight basis. Prior to each destructive harvest each seedling was analyzed for the leaf chlorophyll relative reading (SPAD meter 502, Minolta Inc, USA). The leaves of *Labisia pumila* with different greenness (yellow, light green and dark green) were selected for analysis and total leaf chlorophyll content was analyzed. For each type of leaf greenness, the relative SPAD value was recorded (five points/leaf) and the same leaves sampled for chlorophyll content determination. Leaf disk 3 mm in diameter was obtained from leaf sample using a hole puncher. For each seedling the measurement was conducted on the youngest fully expanded leaves on each plant, generally the second or third leaf from the tip of the stem was used. The leaf disks were immediately immersed in acetone (20 mL) in an aluminum foil-covered glass bottle for approximately 24 hours at 0 °C until all the green colour had bleached out. Finally, the solution (3.5 mL) was transferred to measure at absorbances of 664 and 647 nm using a spectrometer (UV-3101P, Labomed Inc, USA). After that the least squares regression was used to develop predictive relation between SPAD meter readings and pigment concentrations (mg g^−^^1^ fresh weight) obtained from the chlorophyll destructive analysis.

### 3.11. Plant Biomass and Specific Leaf Area Measurements

Total plant biomass was taken by calculating the dry weight of root, stem and leaf per seedling. Plant parts were separated and placed in paper bags and oven dried at 80 °C until constant weight was reached before dry weights were recorded using electronic weighing scale (Mettler-Toledo Model B303-S, Swizerland). Specific leaf area (SLA) were calculated by dividing leaf area of plant (measured using a leaf area meter, LI-3100, Lincoln Inc, USA) divided by total plant biomass [61]. 

### 3.12. Protein Determination

Protein content was determined using method from Bradford [62]. In this method, fresh leaf samples (about 2 g) were cut into pieces using scissor and grinded in mortar with 0.05 M Tris buffer (1 mL, pH 8.5) and make to powder with liquid nitrogen. The homogenate was then centrifuged at 9000 rpm for 10 minutes and then stored at refrigerator at 4 °C for 24 hour. After the extraction, about 100 µL from the samples (supernatant) were added with Bradford reagent (Sigma, St Louis, USA, 3 mL) (prepared by using 10 mL of the reagent diluted with 50 mL distilled water) were added and then were incubated for 5 minutes before measured at 595 nm with spectrophotometer. In this method bovine serum (Sigma, St Louis, USA) was used as a standard to produce calibration curve between actual protein content and spectrophotometer readings. The protein was expressed as mg g^-1^ protein fresh weight.

### 3.13. Statistical Analysis

Data were analyzed using analysis of variance by SAS version 17. Mean separation test between treatments was performed using Duncan multiple range test and standard error of differences between means was calculated with the assumption that data were normally distributed and equally replicated.

## 4. Conclusions

The present work has demonstrated that high levels of CO_2_ are able to change the synthesis of flavonoids and phenolics in *L. pumila*. The production of total flavonoids and phenolics was enhanced as CO_2_ levels increased from 400 to 1,200 µmol mol^−1^. With increasing CO_2_, the photosynthesis rate and total non structural carbohydrate were enhanced. Leaf nitrogen content of plants exposed to high CO_2_ was lower than those plants under ambient condition probably due to low sink strength caused by dilution of nitrogen. The extra carbohydrates that cannot be used for growth are channeled for the production of secondary metabolites. The increase in production of secondary metabolites was manifested with high C/N ratio and low protein content in *L. pumila* seedlings. It was also noted that increase in plant secondary metabolites was accompanied by the reduction in chlorophyll content. High levels of CO_2_ may have a real impact on medicinal values of *L. pumila* seedlings.

## References

[1] IPCC (2010). Climate Change 2010: The scientific Basis. Third Assessment Report of the Intergovernmental Panel on Climate Change (IPCC).

[2] Bryant J.P., Chapin F.S., Klein D.R. (1983). Carbon nutrient balance of boreal plants in relation to vertebrate herbivory. Oikos.

[3] Herms D.A., Matson W.J. (1992). The dilemma of plants: to grow or defend. Quarterly Rev. Biol..

[4] Panuelas J., Estiarte M. (1998). Can elevated CO_2_ affect secondary metabolism and ecosystem function?. Trends Ecol. Evol..

[5] Strack D., Dey P.M., Harbone J.B. (1997). Phenolics metabolism. Plant Biochemistry.

[6] Margna U. (1977). Control at the level of substrate supply–an alternative in the regulation of phenylpropanoid accumulation in plant cells. Phytochemistry.

[7] O’Neill G., Norby R.J., Koch G.W., Mooney H.A. (1996). Litter quality and decomposition rates of foliar litter produced under CO2 enrichment. Carbon Dioxide and Terrestrial Ecosystem.

[8] Mark S.J., Jackson S.B. (2000). Growth responses of *Quercus petraea*, *Fraxinus excelsior* and *Pinus sylvestris* to elevated carbon dioxide, ozone and water supply. New Phytol..

[9] Tisserat B., Herman C., Silman R., Bothast R.J. (1997). Using ultrahigh carbon dioxide levels enhances plantlet growth *in vitro*. Hortechnology.

[10] Stiling P., Cornelissen T. (2007). How does elevated carbon dioxide affect plant herbivore interaction ? A field experiment and meta analysis of CO2 mediated changes on plant chemistry and herbivore performance. Glo. Chang. Biol..

[11] Booker F.L. (2000). Influence of carbon dioxide enrichment, ozone and nitrogen fertilization on cotton (*Gossypium hirsitum* L.) leaf and root composition. Plant Cell Environ..

[12] Kim D.O., Jeond S.W., Lee C.Y. (2005). Antoxidant capacity of phenolics phytochemicals from various cultivars of plums. Food Chem..

[13] Yao L.H. (2004). Flavonoid in food and their health benefits. Plant Food Hum. Nutr..

[14] Ho C.T., Lee C.Y., Hungan M.T. (1992). Phenolics compounds in food and their effects on health (analysis, occurance, and chemistry). J. Amer. Chem. Soc..

[15] Heijnen C.G., Haenan G.R., Vancker F.A., Vijgh W.J., Bast A. (2001). Flavonoids as peroxynitrite scavengers: the role of the hydoxyl groups. Toxicol. In Vitro.

[16] Chun O.K., Kim D.O., Lee C.Y. (2003). Superoxide radical scavenging activity of the major polyphenols in fresh plums. J. Agr. Food Chem..

[17] Atoui K., Mansouri A., Bosku G., Kefalas P. (2005). Tea and herbal infusion: their antioxidant activity and phenolics profile. Food Chem..

[18] Cook N.C., Samman S. (1996). Review: Flavonoid chemistry, methabolysm, cardioprotective effects and dietery sources. Nutr. Biochem..

[19] Jaafar H.Z.E., Mohamed H.N.B., Rahmat A. (2008). Accumulation and partitioning of total phenols in two varieties of *Labisia pumila* Benth. under manipulation of greenhouse irradiance. Acta Hort..

[20] Burkill I.H. (1935). A Dictionary of the Economic Products of the Malay Peninsula.

[21] Rozihawati Z., Aminah H., Lokman N. (2003). Preliminary trials on the rooting ability of *Labisia pumila* cuttings. Malaysia Science and Technology Congress 2003.

[22] Jamia A.J., Ibrahim J., Khairana H., Juriyati H. (2004). Perkembangan Penyelidikan dan Pembangunan Kacip Fatimah.

[23] Norhaiza M., Maziah M., Hakiman M. (2009). Antioxidative properties of leaf extracts of popular Malaysian herb, *Labisia pumila*. J. Med. Plant. Res..

[24] Jaafar H.Z.E., Ibrahim M.H., Por L.S. (2010). Effects of CO_2_ enrichment on accumulation of total phenols, flavonoid and chlorophyll content in two varieties of *Labisia pumila* Benth. exposed to different shade levels. Proceedings of International Conference on Balanced Nutrient Management for Tropical Agriculture.

[25] Ghasemzadeh A., Jaafar H.Z.E., Asmah R. (2010). Elevated carbon dioxide increases contents of flavonoids and phenolics compound, and antioxidant activities in Malaysian Young Ginger (*Zingiber officinale* Roscoe) varieties. Molecules.

[26] Kaufman P.B., Cseke L.J., Warber S., Duke J.A., Brielmanm H.L. (1999). Natural products from plants.

[27] Wink M. (1999). Introduction: Biochemistry, Role and Biotechnology of Secondary Products.

[28] Koricheva J., Larsson S., Haukioja E., Keinanen M. (1998). Regulation of woody plant secondary metabolism by resource availability: hypothesis means by meta-analysis. Oikos.

[29] Felgines C., Texier O., Morand C., Manach C., Scalbert A., Regerat F., Remesy C. (2000). Bioavailability of the flavone naringenin and its glycosides in rats. Amer. J. Physiol. Gastrointest. Liver Physiol..

[30] Amin A.A., Rashad M., El-Abagy H.M.H. (2007). Physiological effects of indole-3-butyric-acid and salicylic acid on growth, yield and chemical constituents of onion plants. J. Appl. Sci. Res..

[31] Wang Y.S.H., Bunce A.J., Maas L.J. (2003). Elevated carbon dioxide increases contents of antioxidant compound in field grown strawberries. J. Agr. Food Chem..

[32] Stutte G.W., Eraso I. (2008). Carbon dioxide enrichment enhances growth and flavonoid content of two *Scutellaria* species. J. Amer. Soc. Hort. Sci..

[33] Tissue D.T., Thomas R.B., Strain B.R. (1997). Atmospheric CO_2_ increases growth and photosynthesis of *Pinus taedea*: A four year field experiment. Plant Cell Environ..

[34] Den-Hertog J., Stulen L., Fonseca E., Delea P. (1996). Modulation of carbon and nitrogen allocation in *Urtica diocia* and *Plantago*
*major* by elevated CO_2_: Impact of accumulation of non-structural carbohydrates and ontogenic drift. Physiol. Planta.

[35] Poorter H., Berkel V., Baxter R., Den-Hertog J., Dijkstra P., Gifford R.M., Griffin K.L., Roumet C., Roy J., Wong S.C. (1997). The effects of elevated CO_2_ on the chemical composition and construction costs of leaves of 27 C3 species. Plant Cell Environ..

[36] Baxter R., Ashenden T.W., Farrar J. (1997). Effects of elevated CO_2_ and nutrient status on growth, dry matter partitioning and nutrient content of *Poa alpinia* var. *vivpara* L. J. Exp. Bot..

[37] Fonseca F., Bowsher C., Stulen I. (1997). Impact of elevated atmospheric CO_2_ and nitrate reductase transcription and activity in leaves and roots of *Plantago major*. Physiol. Planta.

[38] De-souza A.P., Gaspar M., Da-silva E.A., Ulian E.C., Waclawovsky A.J., Nishiyama M.Y., Dos M.R.V., Bucjkeridge M.S. (2008). Elevated CO_2_ increases photosynthesis, biomass and productivity and modifies gene expression in sugarcane. Plant Cell Environ..

[39] Shui Y. C., Feng X., Yan W. (2009). Advances in the study of flavonoids in *Gingko biloba* leaves. J. Med. Plant Res..

[40] Jeyakumar P., Velu G., Rajendran C., Amutha R., Savery M.A.J.R., Chidambam S. (2008). Varied responses of blackgram (*Vigna munga*) to certain foliar applied chemicals and plant growth regulators. Legume Res. Int. J..

[41] Khana W., Prithiviraja B., Smith D.L. (2003). Photosynthetic responses of corn and soybean to foliar application of salicylates. J. Plant Physiol..

[42] Kefeli V.I., Kalevitch M.V., Borsari B. (2003). Phenolics cycle in plants and environment. J. Cell Mol. Biol..

[43] Geiger M., Walch-Piu L., Harnecker J., Schulze E.D., Stitt M. (1998). Enhanced CO_2_ leads to a modified diurnal rhythm of nitrate reductase activity in older plants and a large stimulation of nitrate reductase activity and higher levels of amino acids in higher plants. Plant Cell Environ..

[44] Lambers H. (1993). Rising CO_2_, secondary plant metabolism, plant-herbivore interactions and litter decomposition. Theoretical Considerations. Vegetatio.

[45] Porteaus F., Hill J., Ball A.S., Pinter P.J., Kimbal B.A., Wall G.W., Ademsen F.J., Morris C.F. (2009). Effects of free air carbon dioxide enrichment (FACE) on the chemical composition and nutritive value of wheat grain straw. Anim. Feed Sci. Tech..

[46] Meyer S., Cerovic Z.G., Goulas Y., Montpied P., Demotes S., Bidel L.P.R., Moya I., Dreyer E. (2006). Relationship between assessed polyphenols and chlorophyll contents and leaf mass per area ratio in woody plants. Plant Cell Environ..

[47] Michel H., Klaus K. (2001). The protective functions of caretenoids and flavonoid pigments against excess visible radiation at chilling temperature investigated in *Arabidopsis*. Planta.

[48] Ibrahim M.H., Jaafar H.Z.E., Haniff M., Yusop R. (2010). Changes in the growth and photosynthetic patterns of oil palm (*Elaeis guineensis* Jacq.) seedlings exposed to short term CO_2_ enrichment in a Closed Top Chamber. Acta Physiol. Plant.

[49] Jifon J.L., Wolfe D.W. (2002). Photostnthetic acclimation to elevated CO_2_ in *Phaseolus vulgaris* is altered by growth response to nitrogen supply. Glob. Change Biol..

[50] Nagasubramaniam A., Pathmanathan G., Mallika V. (2007). Studies on improving prodcution potential of baby corn with foliar spray of plant growth regulator. Ann. Rev. Plant Physiol. Plant Mol. Biol..

[51] Gleadow R.M., Evans J.R., McCaffery S., Cavagnaro T.R. (2009). Growth and nutritive value of cassava (*Maribot esculenta* Cranz) are reduced when grown in elevated CO_2_. Plant Biol..

[52] Meyer S., Cerovic Z.G., Goulas Y., Montpied P., Demotes S., Bidel L.P.R., Moya I., Dreyer E. (2006). Relationship between assessed polyphenols and chlorophyll contents and leaf mass per area ratio in woody plants. Plant Cell Environ..

[53] Margna U., Margna E., Vainjarv T. (1989). Influence of nitrogen nutrition on the utilization of L-phenylalanine for building flavonoids in buckwheat seedling tissue. J. Plant Physiol..

[54] Jaafar H.Z.E. (2006). Carbon dioxide enrichment technology for improved productivity under controlled environment system in the tropics. Acta Hort..

[55] Edward J.N. (2008). The effects of trinexapac ethyl and three nitrogen sources on creeping bentgrass (*Agrostis stolonnifera*) grown under three light environments. Master Thesis.

[56] Thayumanam B., Sidasivam S. (1984). Carbohydrate chemistry. Qual. Plant Foods Hum. Nutr..

[57] Tognetti R., Johnson J.D. (1999). The effect of elevated atmospheric CO_2_ concentration and nutrient supply on gas exchange, carbohydrates and foliar phenolics concentration in live oak (*Quercus virginiana* Mill.) seedlings. Ann. For. Sci..

[58] Jaafar Z.E.J., Mohd Hafiz I., Philip E. (2009). Leaf gas exchange properties of three varieties of *Labisia pumila* Benth. under greenhouse conditions. J. Trop. Plant Physiol..

[59] Ibrahim M.H. (2008). Carbon dioxide effects on growth and physiological attributes of oil palm seedlings. Master Thesis.

[60] Idso S.B., Kimball B.A., Hendrix D.L. (1996). Effects of atmospheric CO_2_ enrichment on chlorophyll and nitrogen nutrition concentrations of four sour orange tree leaves. Environ. Exp. Bot..

[61] Gardner F.P.I., Pearce R.B., Mitchell R.L. (1986). Physiology of Crop Plants.

[62] Bradford M. (1976). A rapid and sensitive method for the quantification of microgram quantities of protein utilizing the principle of protein dye-binding. Anal. Biochem..

